# An atypical residue in the pore of *Varroa destructor* GABA-activated RDL receptors affects picrotoxin block and thymol modulation

**DOI:** 10.1016/j.ibmb.2014.10.002

**Published:** 2014-12

**Authors:** Kerry L. Price, Sarah C.R. Lummis

**Affiliations:** Department of Biochemistry, University of Cambridge, Cambridge, CB2 1QW, UK

**Keywords:** Cys-loop, *Varroa*, *Drosophila*, Ligand-gated, Antagonist, GABA, nACh, nicotinic acetylcholine, AChBP, acetylcholine binding protein, GABA, γ-aminobutyric acid, ELIC, *Erwinia* ligand-gated ion channel, GLIC, *Gloeobacter* ligand-gated ion channel, PTX, picrotoxin, ACh, acetylcholine, 5-HT, 5-hydroxytryptamine, RDL, resistant to dieldrin

## Abstract

GABA-activated RDL receptors are the insect equivalent of mammalian GABA_A_ receptors, and play a vital role in neurotransmission and insecticide action. Here we clone the pore lining M2 region of the *Varroa* mite RDL receptor and show that it has 4 atypical residues when compared to M2 regions of most other insects, including bees, which are the major host of *Varroa* mites. We create mutant *Drosophila* RDL receptors containing these substitutions and characterise their effects on function. Using two electrode voltage clamp electrophysiology we show that one substitution (T6′M) ablates picrotoxin inhibition and increases the potency of GABA. This mutation also alters the effect of thymol, which enhances both insect and mammalian GABA responses, and is widely used as a miticide. Thymol decreases the GABA EC_50_ of WT receptors, enhancing responses, but in T6′M-containing receptors it is inhibitory. The other 3 atypical residues have no major effects on either the GABA EC_50_, the picrotoxin potency or the effect of thymol. In conclusion we show that the RDL 6′ residue is important for channel block, activation and modulation, and understanding its function also has the potential to prove useful in the design of *Varroa*-specific insecticidal agents.

## Highlights

• The *Varroa* mite RDL receptor has 4 atypical M2 residues: -7′H, 6′M, 20′S and 21′Q.
• A *Drosophila* T6′M RDL receptor has a decreased GABA EC_50_ and is resistant to picrotoxin block.
• The T6′M mutation ablates thymol enhancement of RDL responses.
• N-7′H, A20′S and A21′Q do not modify GABA, picrotoxin or thymol effects.
• The data could be useful in the design of *Varroa*-specific insecticidal agents.

## Graphical abstract

**Figure:**
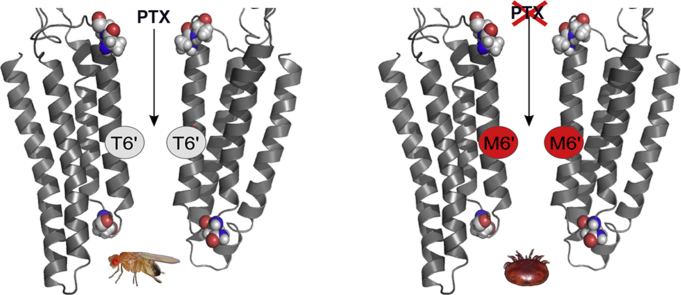

## 1 Introduction

The ectoparasitic mite, *Varroa destructor*, is the primary pest of the honeybee *Apis mellifera*. It is found in large parts of the world and is spreading to others, although is still absent from some areas such as Australia (Rosenkranz et al., 2010). It is a problem because it has significant effects on bees, weakening colonies by introducing viruses such as the deformed wing virus, and is considered a major causal factor in colony collapse disorder (CCD). CCD, pathogens, and a loss of natural habitat are the most likely reasons for the significant decline in honey bees observed in the last decade (Potts et al., 2010), and, as the industry is worth billions of dollars annually, this decline is a serious economic problem. The effect of *Varroa* is usually minimised using synthetic control agents, such as fluvalinate and coumaphos, as well as natural compounds including the essential oil thymol, but these too may have adverse effects on bee health (Waliwitiya et al., 2010, Frost et al., 2013, Zhu et al., 2014). Additionally, *Varroa* resistance to commonly used miticides is becoming an increasing problem (Thompson et al., 2002). Thus the identification of an insecticidal target which could be exploited to control *Varroa* mites but not harm bees could have a significantly beneficial effect.

A well understood insecticidal target is the RDL receptor, a GABA-gated ion channel originally named because an amino acid substitution (A302S) in *Drosophila* RDL causes *r*esistance to *d*ie*l*drin (Ffrench-Constant et al., 1991). The RDL receptor is a member of the Cys-loop ligand gated ion channel superfamily, a class of ion channels that underpin fast synaptic transmission in both vertebrates and invertebrates. Cys-loop receptors are pentameric, with a large extracellular domain (ECD) containing the ligand-binding site, and a transmembrane domain (TMD) made up of four α-helices from each subunit (termed M1-M4); the second α-helix (M2) from each subunit lines the pore.

RDL receptors have been identified in a range of insects including honey bees *Apis mellifera* (Jones and Sattelle, 2006), cat fleas, *Ctenocephalides felis* (Bass et al., 2004), red flour beetles, *Tribolium castaneum* (Jones and Sattelle, 2007), and spider mites, *Tetranychus urticae* (Dermauw et al., 2012). They can be expressed as homomers in *Xenopus* oocytes or *Drosophila* S2 cells and have a distinct pharmacological profile when compared to vertebrate GABA-activated Cys-loop receptors (GABA_A_ receptors); they are not, for example, blocked by the classic competitive GABA_A_ antagonist bicuculline although are, like many Cys-loop receptors, blocked by picrotoxin (Ffrench-Constant et al., 1993). These characteristics are similar to GABA receptors in cultured *Drosophila* neurons (Zhang, 1994) and in cultured honeybee antennal lobe neurons (Dupuis et al., 2010), showing that RDL homomers likely predominate *in vivo*. A number of insecticides, including dieldrin and fipronil, act via interactions in the RDL receptor pore regions (Buckingham et al., 2005, Raymond-Delpech et al., 2005). Here we clone the pore-lining region of a *Varroa* RDL receptor and examine differences in this region between *Drosophila* and *Varroa* RDL receptors using electrophysiology. The full sequence of the *Varroa* RDL receptor is not yet known, thus we have taken the opportunity to investigate the role of these differences in the pore lining region in isolation from the rest of the protein. We also test the impact of these changes on the action of thymol, which has been shown to act as a positive allosteric modulator in RDL and GABA_A_ receptors (Priestley et al., 2003).

## 2 Materials and methods

### 2.1 Materials

All reagents unless stated were from Sigma Aldrich and of the highest obtainable grade.

### 2.2 Cloning of the Varroa receptor M2 region

Genomic DNA was isolated from single mites (kindly sent by Dr Alan Bowman, University of Aberdeen) using a simple proteinase K extraction method. Mites were homogenized in 50 μl extraction buffer (10 mM Tris–HCl pH 8.0, 25 mM NaCl, 1 mM EDTA and 200 μg ml^−1^ proteinase K) with a polypropylene pellet pestle. Samples were incubated at 37 °C for 4 h and then at 85 °C for 10 min. Cellular debris was pelleted by centrifugation and 1 μl samples of the supernatant were used for PCR. A *Varroa destructor* whole genome shotgun (WGS) sequence database (Cornman et al., 2010) was mined using BLAST to identify possible RDL sequences, based on homology to *Drosophila melanogaster*, *Ixodes scapularis* and *Rhipicephalus microplus* RDL sequences (UniProt accession numbers P25123, R9S2B1 and V9ZAE7 respectively). The PCR primers Vde_Ex7_F (5′ GTATCATTTTGGTTGCACCGAAATGC) and Vde_Ex7_R (5′ AACCATGACAAAGCAGGTTCCCAG) were used to amplify the *Varroa* M2 region, which spans exon 7 in *Drosophila* RDL. A second nested reaction using primer Vde_Ex7_F with Vde_Ex7_nest_R (5′ GTAGACGTCGATA GATTTGACGTAAG) was employed for further amplification.

### 2.3 Oocyte Maintenance

*X. laevis* oocyte-positive females were purchased from NASCO (Fort Atkinson, Wisconsin, USA) and maintained according to standard methods. Harvested stage V-VI *Xenopus* oocytes were washed in four changes of ND96 (96 mM NaCl, 2 mM KCl, 1 mM MgCl_2_, 1 mM CaCl_2,_, 5 mM HEPES, pH 7.5), de-folliculated in 1.5 mg ml^−1^ collagenase Type 1A for approximately 2 h, washed again in four changes of ND96 and stored in ND96 containing 2.5 mM sodium pyruvate, 0.7 mM theophylline and 50 mM gentamicin.

### 2.4 Receptor expression

*Drosophila* RDL subunit cDNA (kindly gifted from N.Millar) was subcloned into pGEMHE for oocyte expression as previously described (McGonigle and Lummis, 2010, Millar et al., 1994). Site directed mutagenesis was performed with the QuikChange mutagenesis kit (Agilent, La Jolla, CA). cRNA was transcribed *in vitro* from linearised pGEMHE cDNA template using the mMessage mMachine T7 Transcription kit (Ambion, Austin, Texas, USA). Stage V and VI oocytes were injected with 5 ng cRNA, incubated at 18 °C, and currents recorded 18–24 h post-injection.

### 2.5 Electrophysiology

*Xenopus* oocytes were clamped at −60 mV using an OC-725 amplifier (Warner Instruments, Connecticut, USA), Digidata 1322A and the Strathclyde Electrophysiology Software Package (Department of Physiology and Pharmacology, University of Strathclyde, UK), or using the Roboocyte (MultiChannel Systems). Currents were recorded at 5 kHz and filtered at a frequency of 1 kHz. Micro-electrodes were fabricated from borosilicate glass (GC120TF-10, Harvard Apparatus, Edenbridge, Kent, UK) using a one stage horizontal pull (P-87, Sutter Instrument Company, California, USA) and filled with 3 M KCl. Pipette resistances ranged from 1.0 to 2.0 MΩ. Oocytes were perfused with ND96 at a constant rate of 12 ml min^−1^. Drug application was via a simple gravity fed system calibrated to run at the same rate.

Analysis and curve fitting was performed using Prism v5 (GraphPad Software, San Diego, California, USA). Concentration-response data for each oocyte was normalised to the maximum current for that oocyte. The mean and S.E.M. for a series of oocytes were fitted to the four parameter logistic equation in Prism.

## 3 Results

### 3.1 The *Varroa* M2 region

**Fig. 1:**
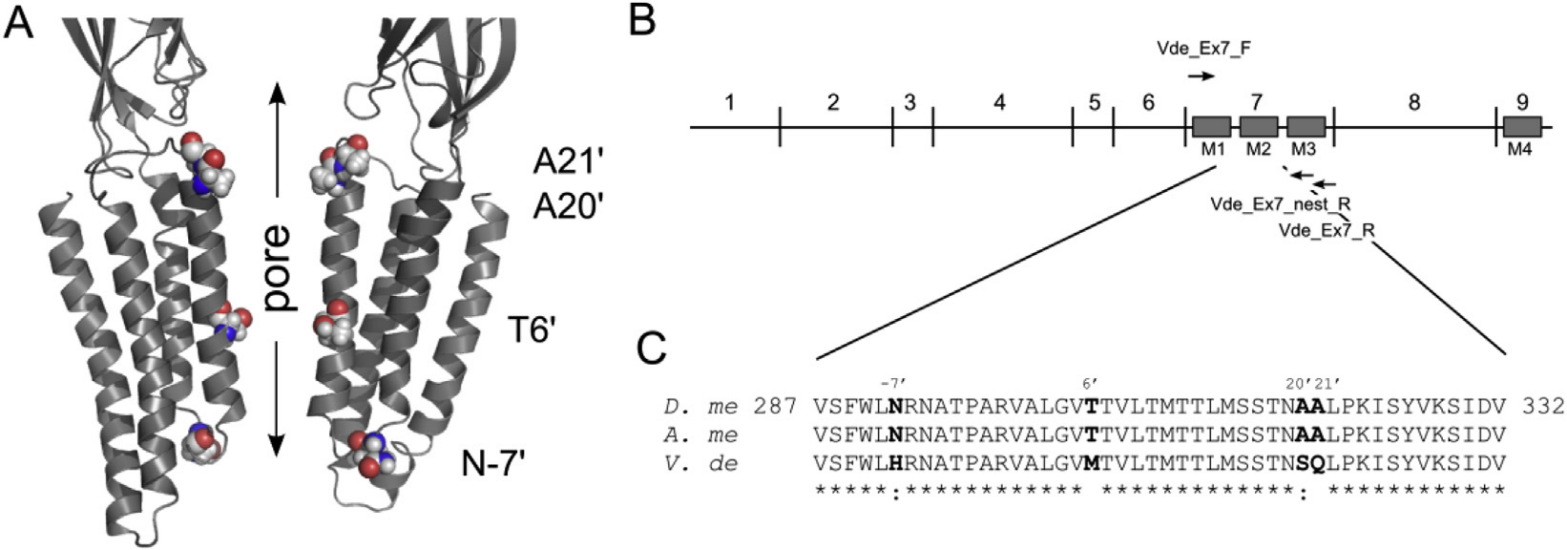
A. Model of the RDL receptor transmembrane region showing two opposing subunits lining the pore. The residues investigated in this study are depicted. B. Exon organisation of the rdl gene denoting the position of the primers used for amplification of the *Varroa* M2 region. C. Sequence of the *Varroa* RDL receptor (*V. De*) amplified by these primers and its alignment with corresponding *Drosophila* (*D. me*) and *Apis* (*A. me*) RDL sequences.

Sequencing of 140 bp PCR products amplified using primers Vde_Ex7_F and Vde_Ex7_nest_R revealed there was a high degree of amino acid sequence homology to the *Drosophila* RDL receptor, but there were 4 different residues: N292H, T305M, A319S and A320Q (Fig 1). Using the prime notation, where the conserved charged residue at the cytoplasmic end of M2 is 0′, these mutations correspond to N-7′H, T6′M, A20′S and A21′Q; this notation will be used for all further discussion. These alternative residues were also identified in the *V. destructor* contig sequence VDK00020745-3167.

### 3.2 RDL activation by GABA

**Fig. 2:**
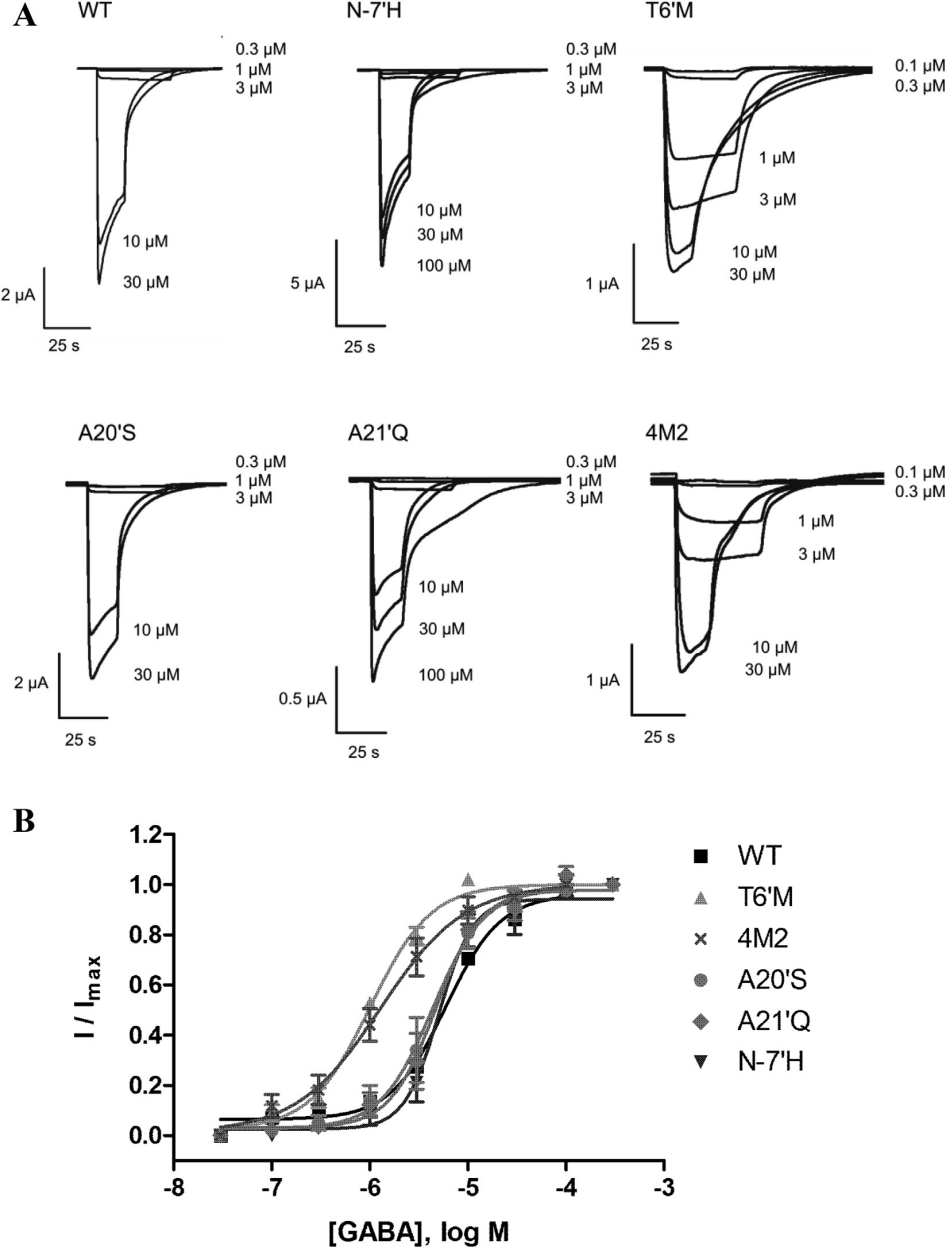
A. Representative current traces from electrophysiological measurements of GABA responses from WT and mutant RDL receptors. GABA was applied for 40 s (0.1–3 μM GABA) or 15 s (10–100 μM GABA). B. Concentration-response curves for activation of RDL receptors by GABA. Parameters obtained from these curves are shown in Table 1.

Application of GABA to *Xenopus* oocytes expressing WT and mutant RDL receptors produced large, reversible inward currents (Fig 2). Plotting current amplitude against a range of GABA concentrations yielded an EC_50_ for WT receptors of 5.9 μM (pEC_50_ = 5.26 ± 0.11) and Hill slope of 1.6 ± 0.5 (Table 1).

**Table 1:** Parameters derived from GABA concentration–response curves at wild type and mutant RDL receptors.

Mutant	GABA			
	pEC_50_ (M)	EC_50_ (μM)	*n*_H_	*n*
Wild type	5.26 ± 0.11	5.9	1.6 ± 0.5	7
N-7′H	5.30 ± 0.05	5.0	2.5 ± 0.5	7
T6′M	6.00 ± 0.04*	1.0	1.4 ± 0.2	8
A20″S	5.36 ± 0.07	4.3	1.6 ± 0.4	6
A21′Q	5.33 ± 0.07	4.7	1.9 ± 0.4	6
4M2	5.89 ± 0.10*	1.3	1.0 ± 0.3	6

Data = mean ± SEM; * significantly different to WT; one-way ANOVA, *p* < 0.01.

### 3.3 Mutant receptors

Mutant *Drosophila* RDL subunits containing the single substitutions N-7′H, T6′M, A20′S and A21′Q were created, as well as a mutant subunit containing all 4 substitutions (4M2). As shown in Table 1, substitution T6′M and 4M2 resulted in a decrease in GABA EC_50_ compared to WT receptors. Substitutions N-7′H, A20′S and A21′Q did not change EC_50_s. Hill coefficients were similar for mutant and WT receptors.

### 3.4 Picrotoxin inhibition

**Fig. 3:**
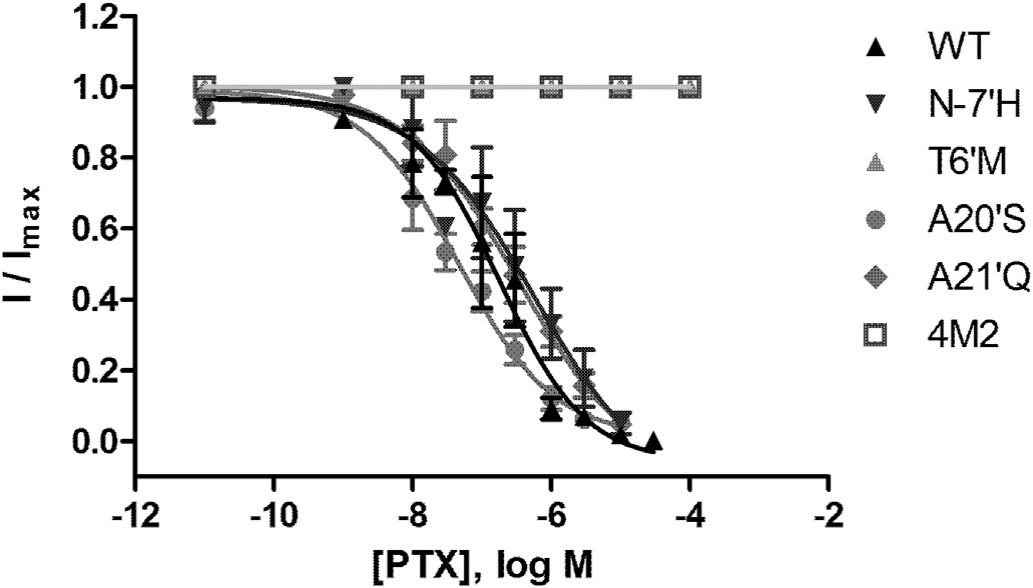
Concentration-inhibition curves for inhibition of GABA responses by picrotoxin. Parameters obtained from these curves are shown in Table 2.

Wild type RDL was inhibited by PTX with an IC_50_ of 180 nM, similar to previous findings (Table 2). T6′M and 4M2 mutants were insensitive to high PTX concentrations (up to 100 μM), while N-7′H, A20′S and A21′Q substitutions did not significantly affect IC_50_s (Table 2, Fig 3).

**Table 2:** Picrotoxin potency at wild type and mutant RDL receptors.

Mutant	pIC_50_ (M)	IC_50_ (nM)	*n*
Wild type	6.75 ± 0.22	180	4
N-7′H	6.25 ± 0.64	560	3
T6′M	NI		6
A20′S	7.33 ± 0.13	46	4
A21′Q	6.45 ± 0.25	360	5
4M2	NI		6

Data = mean ± SEM; NI = no inhibition at 100 μM.

### 3.5 Thymol modulation

**Fig. 4:**
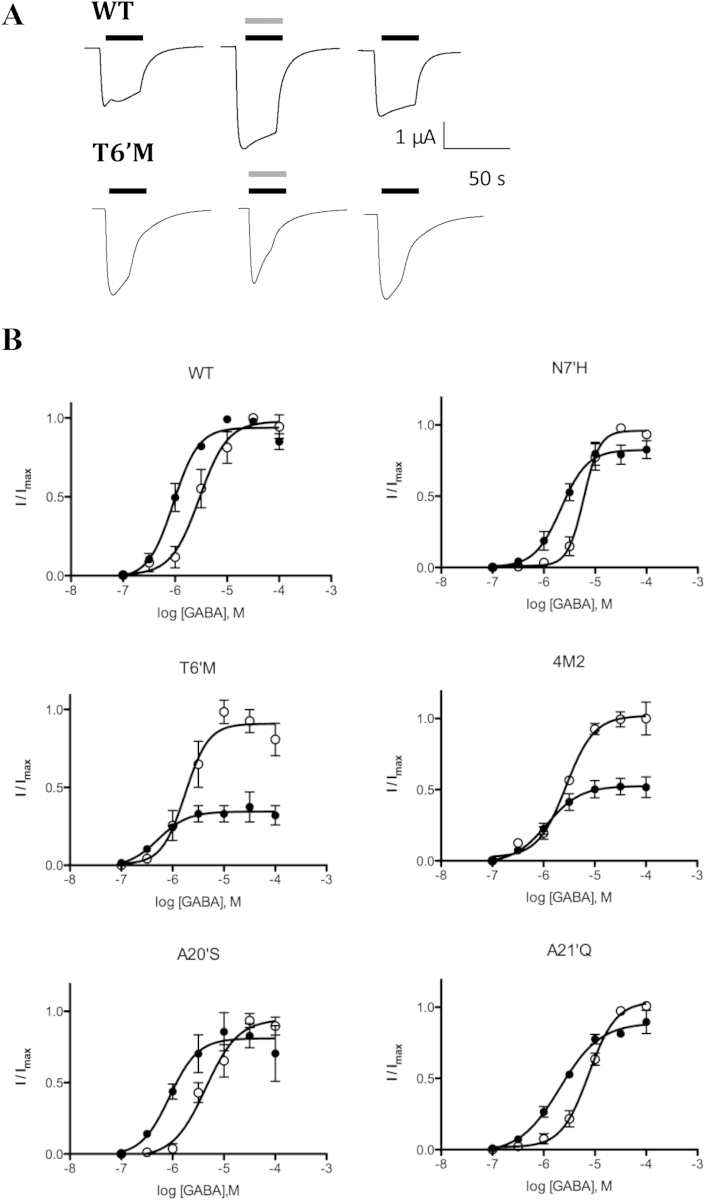
A: Example traces of WT and T6′M RDL receptors activated by 1 μM GABA (black line) in the absence and presence (grey line) of 100 μM thymol. B: GABA concentration–response curves in the absence (open circles) and presence (filled circles) of 100 μM thymol. % *I*_max_ values are WT:94 ± 3; N-7′H:82 ± 4, T6′M:35 ± 9*; 4M2:52 ± 4*; A20′S:81 ± 6; and A21’Q:89 ± 4; * = significantly different to WT, p < 0.05. Other parameters obtained from these curves are shown in Table 3.

Thymol has been previously shown to enhance GABA-induced responses in RDL receptors at 100 μM and similarly did this at our WT, N-7′H, A20′S and A21′Q mutant receptors when using 1 μM GABA. However there was inhibition at some GABA concentrations in T6′M and 4M2 receptors (see example in Fig 4A). Concentration response curves revealed a decrease in EC_50_ in the presence of thymol in WT and all our mutant receptors, but for T6′M and 4M2 RDL receptor there was a concomitant decrease in *I*_max_ (Table 3; Fig 4B)

**Table 3:** Parameters derived from GABA concentration–response curves with and without thymol.

	Control		+Thymol		Fold change in EC_50_
	pEC_50_ (M)	EC_50_	pEC_50_ (M)	EC_50_	
WT	5.36 ± 0.09	4.3	6.03 ± 0.06*	0.9	4.8
A20′S	5.36 ± 0.10	4.4	6.06 ± 0.16*	0.9	5.0
A21′Q	5.12 ± 0.04	7.5	5.68 ± 0.08*	2.1	3.6
N-7′H	5.22 ± 0.05	5.9	5.67 ± 0.08*	2.2	2.7
T6′M	5.76 ± 0.11	1.7	6.28 ± 0.22*	0.5	3.4
4M2	5.74 ± 0.08	1.8	6.20 ± 0.14*	0.6	3.0

*Significantly different to control; one-way ANOVA, *p* < 0.05; Data = mean ± SEM, *n* = 3–4.

## 4 Discussion

GABA-activated RDL receptors are present in the nervous system of insects and likely play a major role in inhibitory responses, similar to the role of GABA_A_ receptors in vertebrates. However RDL receptors have pharmacological characteristics distinct to those of GABA_A_ receptors, which mean they lend themselves to being a target for insecticidally active compounds, examples of which include cyclodienes and fipronil. Such compounds act predominantly in the ion channel, which is constituted from the M2 regions of each of the 5 subunits, and it has long been known that a mutation in this region from A to S at the 2′ position in the *Drosophila* receptor results in resistance to dieldrin (resulting in the name of these receptors). M2 is highly conserved between RDL receptors from different species and thus it was unexpected that the *Varroa* RDL receptor had four atypical residues in this region, one of which is the 6′ pore lining residue which, in both RDL and in other Cys-loop receptors, plays a role in the binding of pore blocking compounds. Characterisation of mutant RDL receptors containing these atypical residues reveals that the 6′ residue is intimately involved in activation, block and modulation, results that likely can be extrapolated to other members of the Cys-loop family. The data also suggest the potential for exploitation in the design of *Varroa* specific insecticides. The residues are discussed in more detail below.

Our data show that T6′M is the most critical of the 4 atypical residues we identified in the *Varroa* M2 region. The 6′ residue lines the pore and has a role in the binding of a range of pore blocking compounds in many Cys-loop receptors, including PTX in GABA_A_ and glycine receptors (Gurley et al., 1995, Shan et al., 2001), lindane and fipronil in glycine receptors (Islam and Lynch, 2012), alcohol in GABA receptors (Johnson et al., 2012) and PTX, ginkgolides and bilobalide in 5-HT_3_ receptors (Das and Dillon, 2005, Thompson et al., 2011). Here we found a T6′M mutation in the *Drosophila* RDL receptor resulted in receptors insensitive to PTX, similar to effects we have previously observed in a T6′V mutation, where PTX and ginkgolide inhibitory effects were ablated (Thompson et al., 2012), and consistent with data from a T6′L mutation found in *Rhipicephalus microplus* (southern cattle tick) RDL receptors which is associated with dieldrin resistance (Hope et al., 2010). The T6′M substitution also decreased the GABA EC_50_ consistent with a role in gating, as has been observed in other Cys-loop receptors; e.g. in 5-HT_3_ receptors a T6′S substitution alters the relative efficacy of a series of agonists, changing some (e.g. quipazine) from apparent antagonists to potent and efficacious agonists (Thompson and Lummis, 2013).

The T6′M mutation also had a significant effect on thymol modulation. Thymol enhances responses in RDL and GABA_A_ receptors, although its mechanism of action is not known. Thymol is one of many modulators of vertebrate GABA_A_ receptors but, despite many decades of study, the molecular mechanisms by which these compounds modulate GABA-induced responses are not yet fully understood (see e.g Amin, 1999; Carter et al., 2010, Sancar and Czajkowski, 2011, Serafini et al., 2000, Smith and Olsen, 1995). Thymol modification shows some similarity to barbiturate modification, as barbiturates can enhance GABA-induced function, and/or directly activate or inhibit receptors, depending on concentration. Barbiturates likely have multiple sites of action, and our data are not inconsistent with multiple sites of action of thymol at RDL receptors: The similar change in EC_50_ in WT and mutant receptors indicates that the mechanism by which thymol exerts this effect is not altered in T6′M receptors, yet the inhibition by thymol differs, suggesting a different mechanism. Combining this with the fact that our data (Fig 4B) suggest the inhibition is non-competitive, it may be that thymol can occlude the channel pore in T6′M receptors, while still acting at a distinct site to enhance the EC_50_. Similarly in GABA_A_ receptors inhibition by short-chain alcohols is mediated by 6′ residues independently of their potentiating effects, which are mediated at structurally distant positions (Johnson et al., 2012, Mihic et al., 1997). More studies are required to identify where and how thymol exerts its effects on GABA_A_ receptors, and on RDL receptors, especially those from *Varroa* when their sequence is known, but the fact that there are differences in thymol effects between wild type RDL receptors and RDL receptors with *Varroa*-like pores, could provide an explanation for its differential effects on bees and mites (Imdorf et al., 1995).

The other residues we identified that differ between *Varroa* and other RDL receptor M2 regions (N-7′H, A20′S, A21′Q) do not have significant effects on function when altered in the *Drosophila* RDL receptor. The N-7′ residue is some distance from the pore, and thus is unlikely to have a major role, as our data suggest. However there has been some interest in the contribution of residues around the 20′ location. Residues at 20′ and 21′ in GABA_A_ receptors have been shown by substituted cysteine accessibility mutagenesis to be water accessible, and therefore probably line the pore at the extracellular end of M2 (Bera et al., 2002). No role in binding pore-blocking compounds or in receptor gating has been identified, but there is some evidence that residues here can contribute to ion flux; e.g. in the cation-selective 5-HT_3_ receptor altering the 20′S residue to a positively charged residue in an E-1′A (non-selective) receptor results in the receptor becoming anion selective (Thompson and Lummis, 2003). The alterations in the *Varroa* sequence do not change the charge profile of this region, but are likely to alter the hydrophobicity: both Ser and Gln are more hydrophilic than Ala, and both have the ability to form hydrogen bonds with compounds that might enter the pore. Wang et al. (1999) studied the N19′ residue in RDL receptors. They found that mutations of this residue caused changes in single channel conductance and reversal potential, though no simple electrostatic model could explain all the data. Thus, while the physiological effect of these atypical residues in *Varroa* RDL receptors await further studies when the full sequence is known, they do provide the possibility of designing compounds that could selectively target *Varroa* and not beneficial insects.

While most RDL receptors identified to date have the same M2 residues as those found in *Drosophila*, this is not true for the M2 region of the spider mite, *Tetranychus* (Dermauw et al., 2012), where 3 variants of RDL have been identified: RDL1 has atypical residues in M2 at 2′ (H) and 6′ (I), whilst RDL2 and RDL3 have 2′S and 6′T. All 3 variants were expressed at all developmental stages, although RDL3 had slightly higher expression levels than the others. The authors observed low sensitivity of spider mites to fipronil, which is likely due to these differences in M2 residues. These variations in M2, and those in *Rhipicephalus* and *Varroa*, may be naturally occurring, but it is also worth considering if they could be the result of selection pressure from the widespread use of insecticides. If this is so, we urgently need to instigate the design of new compounds that target harmful pests. In light of this, data from a recent study are promising: the action of the novel antiparasitic isoxazoline A1443 (fluralaner) were examined on *Tetranychus* RDL receptors expressed in *Xenopus* oocytes and in whole mites (Asahi et al., 2014), and blocked these receptors with an IC_50_ of approximately 10 nM, despite the fact that they were resistant to fipronil. Thus the 2′ and 6′ residues may not be important for the action of fluralaner.

In conclusion we have shown that the amino acid residues that constitute the M2 region in *Varroa* mite RDL receptors differ from those in most other insects in four locations. One of these, the 6′ location, is a major pore blocker binding site, and the *Varroa* 6′ mutation in *Drosophila* receptors ablated PTX inhibition and thymol enhancement. Replacement of the other amino acids in the *Drosophila* M2 region had no significant effect on receptor function, but change the hydrophilicity profile of the pore. Our data indicate that there may be an opportunity to design or identify *Varroa* -specific pore blocking compounds that act at the 6′ and/or the 20′/21′ regions of the RDL receptor pore.
